# Men's values-based factors on prostate cancer risk genetic testing: A telephone survey

**DOI:** 10.1186/1471-2350-5-28

**Published:** 2004-12-10

**Authors:** David J Doukas, Yuelin Li

**Affiliations:** 1 Family and Geriatric Medicine, and Institute for Bioethics, Health Policy, and Law, University of Louisville School of Medicine, Louisville, KY 40202, USA; 2 Center for Outcomes Research, The Children's Hospital of Philadelphia, Philadelphia, PA 19104, USA; 3 Anesthesia, University of Pennsylvania School of Medicine, Philadelphia, PA 19104, USA

## Abstract

**Background:**

While a definitive genetic test for Hereditary Prostate Cancer (HPC) is not yet available, future HPC risk testing may become available. Past survey data have shown high interest in HPC testing, but without an in-depth analysis of its underlying rationale to those considering it.

**Methods:**

Telephone computer-assisted interviews of 400 men were conducted in a large metropolitan East-coast city, with subsequent development of psychometric scales and their correlation with intention to receive testing.

**Results:**

Approximately 82% of men interviewed expressed that they "probably" or "definitely" would get genetic testing for prostate cancer risk if offered now. Factor analysis revealed four distinct, meaningful factors for intention to receive genetic testing for prostate cancer risk. These factors reflected attitudes toward testing and were labeled "motivation to get testing," "consequences and actions after knowing the test result," "psychological distress," and "beliefs of favorable outcomes if tested" (α = 0.89, 0.73, 0.73, and 0.60, respectively). These factors accounted for 70% of the total variability. The domains of motivation (directly), consequences (inversely), distress (inversely), and positive expectations (directly) all correlated with intention to receive genetic testing (p < 0.001).

**Conclusions:**

Men have strong attitudes favoring genetic testing for prostate cancer risk. The factors most associated with testing intention include those noted in past cancer genetics studies, and also highlights the relevance in considering one's motivation and perception of positive outcomes in genetic decision-making.

## Background

There are several factors to consider in undergoing genetic testing for cancer risk: potential benefits, possible risks, psychological distress, and the uncertainty in subsequent decision-making about prophylactic interventions [1-9]. While the health professional's assessment of the potential benefits and harms frames the disclosure of informed consent, the patients' values and expectations are intrinsic on the decision-making process. Current understanding of these values and expectations has been primarily derived from patients considering genetic testing for breast and colorectal cancers [10]. It remains unclear how these same factors may influence men's decision making in testing for hereditary prostate cancer risk [2]. The question addressed in this article is what values and expectations influence the intention of men to undergo genetic testing for prostate cancer risk.

A definitive genetic test for prostate cancer is not clinically available yet. Current genetic tests are only conducted in research studies. Several potential genetic loci have been identified as linked to hereditary prostate cancer, including HPC1 [11], MXI1, KAI1, [12] and 1q42.2-q43 on chromosome 1q. In the future, a test (or set of tests) for hereditary prostate cancer risk may become available. Such testing may become an important tool in preventing prostate cancer, or be useful once prostate cancer is diagnosed (e.g., for treatment decisions). Further, it is prudent for physicians to be prepared for patient requests for genetic testing, even when there are no strong clinical indications. Learning why men would accept or refuse prostate cancer genetic risk information is therefore relevant to the future of testing, and its informed consent.

Informed consent for genetic testing for cancer risk is particularly controversial in cancers where knowledge of a positive test result does not provide opportunities for interventions for favorable outcomes, and a negative result does not provide reassurance [13]. High stated intention for genetic testing for prostate cancer risk (over 80%) has been reported in the past [14]. Identification of a man at genetic risk for prostate cancer presents an ambiguous dilemma: Should a positive result be followed by prophylactic surgery, medication, increased surveillance (via PSA testing or rectal examination), or standard screening recommendations? The knowledge gained through genetic screening may not necessarily lead to clear cut recommendations about what the patient should do next.

This study examines men's beliefs and values toward interest in prostate cancer genetic testing. A survey instrument was developed for men between 40 and 70 years of age, exploring their beliefs, attitudes, and concerns in considering a hypothetical blood test. Exploratory factor analysis was applied to identify the underlying factor dimensions. The relative importance of these factors was then compared to testing intention.

## Methods

### Study population

The Institutional Review Board (I.R.B.) of the University of Pennsylvania and US Department of Defense Human Subjects Review approved the study. Subjects for this study included healthy outpatient males, identified with the assistance of the institution's Office of Health Services Research for demographic characteristics of age, ethnicity, and absence of past or current history of prostate cancer. Subjects were sent a letter-invitation to participate with an opt-out telephone number to call. Inclusion criteria were that subjects must be English-speaking men in a large metropolitan East-coast city, between the ages of 40 and 70, with no current evident mental incapacity and no present or past personal history of prostate cancer. All others were excluded.

### Prostate cancer genetic screening survey questionnaire

#### Survey development

A 53-item attitude survey instrument was developed. The items were selected by the collaborators from a pool of more than 100 preliminary items from the data resulting from 12 focus groups of 90 lay men regarding their attitude, beliefs, and concerns about prostate cancer genetic screening [15]. The statements were answered on a 1–5 Likert-type scale ("Strongly Disagree" = 1, "Disagree" = 2, "Neutral" = 3, "Agree" = 4, and "Strongly Agree" = 5). Twenty-one items were reverse phrased to counter balance directionality in the response scale. Items 1, 51, 52, and 53 were intent items: "I would want the genetic test for prostate cancer risk when it becomes available," "I would want this test if it could tell me that prostate cancer is more likely to happen earlier in my life," "I would want this test if it could tell me that prostate cancer is more likely to be more life threatening because I have the prostate cancer risk gene," and "I would want this test even if it does not tell me new information about how early or aggressive prostate cancer may be in my future," respectively.

### Telephone interview

The survey was conducted using Computer-Assisted Telephone Interviewing software (MacCATI, Senecio Software). The survey instrument was pilot tested in face-to-face interviews of randomly selected men, age 40–70, in a primary care office prior to data collection, to verify understandability of the survey's content and format. For the telephone survey, a recruitment packet that included an informed consent letter was first mailed to the prospective participants. Instructions explained the goals of the study and gave them an option to opt-out with a toll-free phone call prior to their interview. An oral informed consent was completed prior to the telephone interview.

### Missing data

The number of missing observations ranged from 0 to 13, with an average of 2.38. Missing data were imputed based on an imputation model that predicts the missing values of factors as predicted by all of the other responses, including the outcome (desire to be tested). The algorithm uses Markov Chain Monte Carlo methods to select at random a value from the distribution of the possible values predicted by the missing value model. This method differs in several respects from other methods of filling in for missing data, in that with each imputation a different value will be imputed for the missing value, thus ensuring an added dimension of variability in the resulting analyses. The imputation was repeated multiple times. Each imputation generated an imputed data set. The same factor analysis was applied and no statistically reliable differences were found across the imputed data sets. Thus, only the results from the first imputed data are reported here. The imputation was carried out by SAS PROC MI.

### Factor analysis and reliability statistics

A maximum-likelihood factor analysis with oblique rotation was applied to the 49 non-intent questions to classify men's non-intent beliefs and attitudes according to their underlying dimensions. The four questions that directly probed men's expressed intent were considered *a priori *as a separate factor. The factor analysis involved methodological criteria for data reduction, which included the rules summarized in Tabachnick [16]. Items with factor pattern loading lower than .40 were dropped (less than 16% overlapping variance between the item and the associated factor). The most salient dimensions were then retained, accounting for at least 70% of the total variability. The internal consistency reliability was assessed by Cronbach's alpha coefficient [17]. Items that showed the highest factor pattern loadings for a particular factor were considered items that measure the attitude associated with that dimension [18]. Factor scores, with estimated scores on each of the individual factors had they been measured directly, were also derived by summing the raw scores of the items [19].

## Results

### Demographics

Interviews were completed with 400 respondents with a cooperation rate of 47% (1675 were contacted, 431 refused to participate either by phone prior to the interview, or at the time of the interview, and 844 were excluded due to no answer, disconnected telephones, and death). Table 1 summarizes the respondents' characteristics. Of note, another study by the authors revealed that no demographic factor had a moderating impact on intention, except one – in which higher levels of education correlated with diminished testing intention [20]. IRB constraints precluded non-respondent data collection for comparison.

**Table 1:** Respondent characteristics

Characteristics	N (N = 400)	%
Ethnicity		
White	288	72
Black	87	22
Hispanic	5	1
Asian	8	2
Other	6	2
No response	6	2
Age		
40–49	133	33
50–59	143	36
60–69	124	31
Education		
< High school	86	22
High school graduate/some college	149	37
College graduate	141	35
Post-graduate degree	23	6
No response	1	0
Annual household income		
$15,000 or less	17	4
$15,001 – $45,000	74	19
$45,000 – 75,000	84	22
$75,000 – $105,000	90	23
More than $105,000	107	28
No response	28	7
Marital status		
Married	319	80
Steady relationship but not married	23	6
Separated or divorced	26	7
Single	25	6
Widowed	7	2

### Testing intention

About 82% of men interviewed expressed that they "probably" or "definitely" would take the test if one were offered now. This high interest increased to 88% if a positive test result indicates elevated risk in the early onset of cancer; 93% if it indicates graver prognosis of cancer; and the stated interest dropped to a still appreciable 68% if *no new information *on timing or severity of prostate cancer is to be learned from the prospective test.

### Subscales

Exploratory factor analysis identified four underlying factors that accounted for 76% of the total variability among the 49 items probing men's beliefs and attitudes. The four factors were 1) *Motivation*, i.e., those values relating how strongly the respondent wanted the test, and how strongly the opinions of professionals, spouse, family, relatives, and friends could have influenced the respondent's own strength of intent; 2) *Consequences*, which measured beliefs with respect to follow-up decision-making and management; 3) *Distress*, which assessed fear of losing health and life insurance, anxiety, and worsening of quality of life if tested positive; and 4) *Positive Expectations*, which described beliefs in how the test results will confer useful information in family risk and favorable outcomes. The four intent items were added separately as the fifth subscale 5) *Intention *directly probing the respondent's stated intent. Table 2 summarizes the subscales, their respective internal consistency, and the factor loadings of their constituent items.

**Table 2:** Subscales, internal consistency, and factor item loadings

**Factors / statements (internal consistency statistics)**	**Factor pattern loading**
*Motivation*	
Subscale 1: Motivation (alpha = 0.89, 37% variability)	
Even if other relatives did not want me to, I would get genetic testing.	0.80
Even if my children did not want me to, I would get genetic testing.	0.77
I would get genetic testing if my friends wanted me to.	0.73
Even if my friends did not want me to, I would get genetic testing.	0.72
I would get testing if other relatives wanted me to.	0.68
Even if my wife or partner did not want me to, I would get genetic testing.	0.68
Even if a genetic testing specialist recommended against it, I would get genetic testing.	0.60
I would get testing if my children wanted me to.	0.59
Even if my doctor recommended against it, I would get genetic testing.	0.57
I would get testing if a genetic testing specialist recommended it.	0.46
I would get testing if my wife or partner wanted me to.	0.43
*Consequences*	
Subscale 2: Consequences and actions after knowing the test result (alpha = 0.73, 23% variability)	
I find that my concerns about getting prostate cancer interfere with my every day life. [R]	0.56
I don't want testing unless there is a prostate cancer cure. [R]	0.54
If I know I have the prostate cancer risk gene, it will make me feel guilty. [R]	0.53
I'll have to make a quick treatment decision if I know I have the prostate cancer risk gene. [R]	0.52
If I know I have the prostate cancer risk gene, I will make me want to end my life. [R]	0.51
If I don't have the prostate cancer risk gene, I will be able to put my mind at rest about prostate cancer. [R]	0.51
I don't want testing unless it can tell me whether I have prostate cancer now. [R]	0.49
I would not want to have children if I know I have the prostate cancer risk gene. [R]	0.44
I would want to put off testing as long as I can.	0.42
*Distress*	
Subscale 3: Psychological distress (alpha = 0.73, 10% variability)	
I am concerned I will lose or not be able to get LIFE insurance if I get the genetic testing for prostate cancer risk. [R]	0.64
If I know I have the prostate cancer risk gene, it will make me anxious. [R]	0.59
I am concerned I will lose or not be able to get HEALTH insurance if I get the genetic testing for prostate cancer risk. [R]	0.57
If I know I have the prostate cancer risk gene, I will feel worse about myself. [R]	0.46
My life will get worse if I know I have the prostate cancer risk gene. [R]	0.46
If I know I have the prostate cancer risk gene, it will change the way I think about the future.	0.45
*Positive expectations*	
Subscale 4: Beliefs in favorable outcomes if tested (alpha = 0.60, 7% variability)	
I believe this test could save my life.	0.52
The more I know about my risk for prostate cancer, the better I will feel about testing.	0.51
The test results might provide valuable information on prostate cancer risk to my family members.	0.45
If I know I have the prostate cancer risk gene, my doctor may want to do more tests.	0.42
*Intent*	
Subscale 5: Intention (alpha = 0.79)	
I would want the genetic test for prostate cancer risk when it becomes available.	-
I would want the test if it could tell me that prostate cancer is more likely to happen earlier in my life.	-
I would want this test if it could tell me that prostate cancer is more likely to be more life threatening because I have the prostate cancer risk gene.	-
I would want this test even if it does not tell me now information about how early or aggressive prostate cancer may be in my future.	-
[R] – Item reversed in coding for analysis N.B. – Intent items were not included in the factor analysis, thus there are no available data on pattern loadings and variance.	
*The following factors did not load onto the four value-based factor domains, and were omitted from further analysis:*	
I want to wait on testing until it is shown to be very accurate. I will not be able to keep my job, or get a promotion, if I know I have the prostate cancer risk gene. If I know I have the prostate cancer risk gene, my doctor might pressure me to receive treatment. Nothing can be done to prevent prostate cancer. Changes in my lifestyle can reduce my risk of cancer. I only want my family doctor to do this test for prostate cancer risk. I don't want testing unless I can do something to prevent prostate cancer. The government could use my test results in ways I do not want. I often worry about getting prostate cancer If I know I do not have the prostate cancer risk gene, I won't need rectal exams or PSA tests as often. No matter my results, I would want testing if it helps find a cure. I don't want the test if my health care coverage does not pay for it. I would want to get tested because I just want to know if I have the gene for prostate No one should give out my test results to anyone else without my permission. I would get testing if my doctor recommended it.	

Overall, the five subscales showed satisfactory internal consistency. The four items within Intention scale, although grouped together *a priori *for their content, showed a good internal consistency alpha coefficient at 0.79. Among the 49 items that probed men's beliefs, values, and attitudes, the 11 items that loaded high on Motivation accounted for most of the variability (37%) with a very high alpha coefficient (0.89). The nine items in Consequences accounted for the next largest amount of variability (23%) with an alpha coefficient of 0.73. The six items in Distress accounted for 10% of the variability with an alpha of 0.73. Finally, the four items in Positive Expectations accounted for 7% of the variability with an alpha of 0.60.

The factor pattern loadings reflect the correlation between an individual item and its subscale. For example, Table 2 shows that Motivation is strongly associated with the item "even if other relatives did not want me to, I would get genetic testing." (Loading value = 0.80). Respondents with high motivation tended not to be influenced by other relatives. Importantly, the less one is influenced by a relative's opinion, the more likely he is to be motivated to get testing. Conversely, a man who was easily influenced by his spouse or children was somewhat less likely to be motivated toward testing. This latter set of values may reflect the desire for more information and counsel.

The inter-correlations between the subscales are summarized in Table 3, and reveal how these subscales were associated with one another and how they affected intent. The respondents' motivation (regarding the influence of others in their decision) was positively correlated with intention to test (*r *= 0.69, *p *< 0.001). There was also a positive and statistically significant correlation between one's motivation and one's expectations that genetic screening may lead to favorable outcomes for the gene carrier and his family (*r *= 0.39, *p *< 0.001). Concerns about the consequences of a positive result, including the uncertainties of test validity and accuracy, and the availability of subsequent interventions, were positively correlated with distress (*r *= 0.34, *p *< 0.001) and diminished intention to test (*r *= -0.16, *p *< 0.01). Distress-based values were associated with diminished intention to test (*r *= -0.17, *p *< 0.001). Finally, respondents who expected favorable outcomes were associated with increased intention to test (*r *= 0.48, *p *< 0.001).

**Table 3:** Inter-correlations between subscales

	**Motivation**	**Consequences**	**Distress**	**Positive Expectations**
Motivation	-			
Consequences	-0.08	-		
Distress	-0.19*	0.34*	-	
Positive Expectations	0.39*	-0.02	-0.003	-
Intention	0.69*	-0.16*	-0.17*	0.48*

* p < 0.001

## Discussion

These data demonstrate that men in the general public, aged 40 to 70 years without a personal history of prostate cancer, consider prostate cancer genetic testing related to four value-based factor domains, similar to past literature findings on genetic testing for hereditary cancer risk. The motivation factor, which measures values of influence by others, is the strongest decision factor in guiding their opting for the test. More than 80% of men interviewed would consider getting tested if the test was available now. Their stated intention, as measured by the four intent items, is highly correlated with how strongly they feel they are motivated toward the test and inversely related to family influences. Men with strong motivation to get tested also have significantly lower concerns about psychological distress and higher levels of positive expectations. The recommendations of physicians and geneticists are important to men's expressed motivation, although the professionals did not appear to be more influential than their kin.

A respondent is more likely to want the test if he believes that the test may be informative of *family risk *and may lead to early identification and prevention of cancer (as part of the Positive Expectations domain). The influences of kin, along with beliefs in family risk, highlight the importance of reviewing family-related risk information as part of genetic consultation and informed consent. Men undergoing informed consent for hereditary prostate cancer risk in the future not only should be provided information on what genetic testing can and cannot do for them, but also what the test results could mean for others surrounding them (as evidenced by the influences of family, etc.).

Prior hereditary breast cancer (BRCA) and colorectal cancer (CRC) literature has noted anecdotally that perception of benefit to one's family influences genetic test uptake. Eliciting patient perceptions of concerns regarding their family may be beneficial to consider in oncology genetic testing generally. Similar to this literature, intention was found to be influenced by the respondent's concerns about test validity, test accuracy, and by the availability of interventions that may lead to favorable outcomes. Not surprisingly, men who were concerned about potential psychological distress were less likely to want the test. One unanswered question is how men's anticipatory distress and expected adverse consequences may affect how family risk information is interpreted and discussed. Few men in our study anticipated high levels of distress. Although literature data clearly show elevated distress among patients and their family members [21]. More research is needed to better establish the family-risk construct and how it may be influenced by other beliefs and values.

The present study has limitations. Given the exploratory nature of factor analysis, these data are aimed at identifying coherent subsets of variables for data reduction, not at identifying specific attitude statements that discriminate skeptics from supporters. Nevertheless, the reduced set of 34 items is the most important among the administered 57 items, and comprises a coherent and reliable assessment tool of eliciting values and intention toward testing. This item set can thereby serve as a foundation for a confirmatory health beliefs model, using Structural Equation Modeling techniques to better elucidate the interactions of these value-based domains [22]. Also, we noted that this population had somewhat higher income and education levels than the overall Philadelphia Consolidated Metropolitan Statistical Area (CMSA). 51% of men had over $75,000 income, compared with the 32% in the Philadelphia CMSA 2000 census year dataset, and 41% had completed a Bachelor's degree or higher, compared with 28% in the CMSA. These differences may be due to affluent subjects living in suburban counties in the metropolitan Philadelphia area, who then self-select to be seen by physicians in the University of Pennsylvania system. As noted above, our prior work demonstrated no demographic differences except education (with more education correlating with diminished intention). Thus, we do not foresee an adverse impact of these discrepancies on the overall outcomes of our analysis [20].

Future directions of this research may include exploring the relationship between stated intent in prostate cancer genetic screening and actual testing behavior when testing is available. Studies have shown that expressed intention does not necessarily translate to actual behavior in taking genetic tests for breast and colorectal cancers [10,23-29]. The same discrepancy between attitude and behavior may exist when a test for prostate cancer is available for the general public. Our data suggest that potential psychological distress, worries about test validity, insurance, confidentiality, and the uncertainties in subsequent intervention decisions may need to be balanced with family considerations when testing becomes available [30].

## Conclusions

Men in this survey voiced strong attitudes favoring future genetic testing for prostate cancer risk. In the past decade and a half, genetic testing for a variety of cancers concentrated on several key concepts: i.e., stigmatization, privacy, anxiety/stress, and the need to know. These notions of stigma and psychological impact were not as relevant in this population regarding prostate cancer risk genetic testing. For examples, the following statements did not show strong enough factor loadings to warrant their inclusion, such as "I will not be able to keep my job, or get a promotion, if I know I have the prostate cancer risk gene," "The government could use my test results in ways I do not want," "I often worry about getting prostate cancer," and "I would want to get tested because I just want to know if I have the gene for prostate cancer."

The most relevant aspect of data reported herein is that they begin to shed new light on the relevance of "others." How men were concerned about the impact on and the effects upon one's family were reflected in the factor analysis. As a result, future informed consent may likely include considerations of 1) how the test results will affect their own future lives, and 2) how the test results will affect their family members. The latter consideration is seldom brought into the informed consent process in the genetic counseling but may be relevant to the patient.

## Competing interests

The author(s) declare that they have no competing interests.

## Authors' contributions

DD designed the overall project study, collaborated in the data analysis, and participated in its coordination. YL designed and carried out the factor analysis statistics. Both authors drafted, read, and approved the final manuscript.

## Pre-publication history

The pre-publication history for this paper can be accessed here:


